# Detection of human neurotropic JCPyV DNA sequence in pediatric anaplastic xanthoastrocytoma

**DOI:** 10.1007/s13365-023-01129-z

**Published:** 2023-04-25

**Authors:** Sara Passerini, Carla Prezioso, Annalisa Prota, Giulia Babini, Lavinia Bargiacchi, Daniela Bartolini, Ugo Moens, Manila Antonelli, Valeria Pietropaolo

**Affiliations:** 1 Department of Public Health and Infectious Diseases, “Sapienza” University of Rome, P.1e Aldo Moro, 5, 00185 Rome, Italy; 2 Laboratory of Microbiology of Chronic-Neurodegenerative Diseases, IRCCS San Raffaele Roma, Rome, Italy; 3 Department of Radiological, Oncological and Anatomo-Pathological Sciences, “Sapienza” University of Rome, Rome, Italy; 4 Pathology Unit, Bufalini Hospital, Cesena, Italy; 5 Department of Medical Biology, Faculty of Health Sciences, University of Tromsø, The Arctic University of Norway, Tromsø, Norway

**Keywords:** Xanthoastrocytoma, JCPyV, LTAg, Transformation, Rb, NCCR

## Abstract

Due to its peculiar histopathological findings, pleomorphic xanthoastrocytoma (PXA), a rare cerebral tumor of young adults with a slow growth and a good prognosis, resembles to the lytic phase of progressive multifocal leukoencephalopathy, a fatal neurodegenerative disease caused by JC polyomavirus (JCPyV). Therefore, the presence of JCPyV DNA was examined in an 11-year-old child with xanthoastrocytoma, WHO grade 3, by quantitative PCR (qPCR) and nested PCR (nPCR) using primers amplifying sequences encoding the N- and C-terminal region of large T antigen (LTAg), the non-coding control region (NCCR), and viral protein 1 (VP1) DNA. The expression of transcripts from *LTAg* and *VP1* genes was also evaluated. In addition, viral microRNAs’ (miRNAs) expression was investigated. Cellular p53 was also searched at both DNA and RNA level. qPCR revealed the presence of JCPyV DNA with a mean value of 6.0 × 10^4^ gEq/mL. nPCR gave a positive result for the 5ʹ region of the *LTAg* gene and the NCCR, whereas 3ʹ end LTAg and VP1 DNA sequences were not amplifiable. Only LTAg transcripts of 5ʹ end were found whereas *VP1* gene transcript was undetectable. Although in most cases, either Mad-1 or Mad-4 NCCRs have been identified in association with JCPyV-positive human brain neoplasms, the archetype NCCR structure was observed in the patient’s sample. Neither viral miRNA miR-J1-5p nor p53 DNA and RNA were detected. Although the expression of LTAg supports the possible role of JCPyV in PXA, further studies are warranted to better understand whether the genesis of xanthoastrocytoma could depend on the transformation capacity of LTAg by Rb sequestration.

## Introduction

JC polyomavirus (JCPyV) is a ubiquitous human virus isolated in 1971 from the brain of a patient with Hodgkin disease (Padgett et al. 1971). It is the etiological agent of the progressive multifocal leukoencephalopathy (PML), a demyelinating disease of the brain, caused by lytic infection of oligodendrocytes upon viral reactivation (Pietropaolo et al. 2018). The viral genome comprises the early and late gene region and the non-coding control region (NCCR). The early region encodes for nonstructural proteins, large T antigen (LTAg), small t antigen (stAg), and T’135, T’136, and T’165 proteins involved in the regulation of the virus cycle and in cell transformation (Frisque et al. 1984; Khalili 2001). The N-terminal region of LTAg can interact with members of the retinoblastoma (Rb) protein family, whereas the C-terminal domain can bind p53 (Zheng et al. 2022). The interaction with these tumor suppressors induces progression of the cell cycle and is a major feature of the oncogenic properties (Del Valle et al. 2001). The late region encodes for the capsid proteins VP1, VP2, and VP3, for the agnoprotein (Frisque et al. 1984), and for two mature microRNAs (miRNAs), miR-J1-3p and miR-J1-5p, which can modulate viral replication by downregulating LTAg expression (Giovannelli et al. 2015). The NCCR, encompassing the origin of replication and transcription control sequences, has a hypervariable structure that contributes to neurotropism and neurovirulent properties of JCPyV (Pietropaolo et al. 2018). The archetype NCCR structure has been isolated in the kidney and urine from healthy subjects (Yogo et al. 1990) whereas the rearranged or “prototype” strain is frequently disease-associated (Frisque et al. 1984). JCPyV-mediated oncogenesis has been described both in in vitro and in vivo studies (White and Khalili 2005). Inoculation of JCPyV into rodents, owl, or squirrel monkeys produces central nervous system (CNS) tumors including medulloblastoma, astrocytoma, glioblastoma, and neuroblastoma **(**Khalili 2001; Del Valle and Khalili 2021). JCPyV DNA was also detected in human brain tumors such as glioblastomas, astrocytomas, and medulloblastomas (Ahye et al. 2020).

In 1998, JCPyV DNA with a Mad-4 type NCCR was detected in the brain tissue of a 9-year-old child with pleomorphic xanthoastrocytoma (PXA) (Boldorini et al. 1998). So far, no additional cases of JCPyV-positive PXA have been reported.

## Case presentation

**Table 1:** Summary of JCPyV viral load, DNA sequences, LTAg/VP1 transcripts, and miRNA detected in tumor tissue

**Tumor type**	**Age, gender**	**Viral load (gEq/mL)**	**LTAg**		**NCCR**	**VP1**	**LTAg transcripts**		**VP1 transcript**	**JC-miR-5p**
			***5'***	***3'***			***5'***	***3'***		
Anaplastic xanthoastrocytoma (PXA) (gr. III WHO 2021)	11, M	6.0 × 10^4^	+	-	Archetype	-	+	-	-	-

*gEq/mL* genome equivalents/milliliter

An 11-year-old child (Table 1) was admitted to the hospital in January 2022 with a right temporo-parietal mass on magnetic resonance scan images. Pathology slides revealed an anaplastic PXA (WHO grade III). Increased cellularity, mitotic index (7 mitoses/10 hpf), and necrotic foci were evident. Anaplastic features, such as big nucleolus and an abundant eosinophilic cytoplasm absorbed by a fibrillary stroma, were displayed by the cells, and some of these showed big and pleomorphic nuclei, nuclear pseudo inclusions, and foamy cytoplasm. Pseudo-papillary and fascicular growth areas and eosinophilic granular bodies were also observed.

**Fig. 1:**
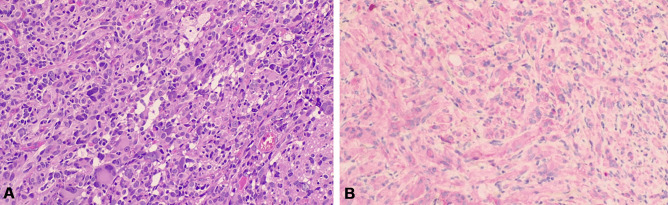
Brain histopathology findings. **A** Pleomorphic tumor cells with occasional bizarre-looking nuclei and admixture of spindle neoplastic and neoplastic cells with bizarre nuclei or multi-nucleation. The neoplastic cells show multi-vacuolated cytoplasm. Granular cell bodies are also evident. **B** BRAFV600E immunohistochemical stain highlights pleomorphic tumor cells

Immunohistochemical analysis showed diffuse positivity for glial fibrillary acid protein and patchy positivity for synaptophysin; however, positivity for mutated BRAFV600E protein has also been demonstrated (Fig. 1).

Total DNA was extracted from the paraffin-embedded tissue sections using Quick-DNA FFPE Miniprep (Zymo Research, Irvine, CA) and was evaluated for its PCR suitability by amplifying the *β-globin* gene sequences. To detect the presence of JCPyV DNA, a quantitative PCR (qPCR), targeting LTAg DNA sequence, was performed. JCPyV DNA was further subjected to nested PCR (nPCR) using primers mapping sequences encoding the N- and C-terminal region of LTAg, the NCCR, and the VP1 gene (Flaegstad et al. 1991; Krynska et al. 1999). Positive PCR products were purified using miPCR purification kit (Metabion, Planegg, Germany) and sequenced in a dedicated facility (Bio-Fab Research, Roma, Italy). In addition, the expression of transcripts from JCPyV *LTAg* and *VP1* genes was investigated. Total RNA was extracted using Quick-RNA Miniprep Plus Kit (Zymo Research, Irvine, CA), reverse transcribed by ZymoScript RT PreMix Kit (Zymo Research, Irvine, CA), and used for a PCR carried out with the same primers used on DNA. In order to investigate JCPyV miRNA expression, the reverse-transcribed RNA was used for PCR amplification of miR-J1-5p coding region (Giovannelli et al. 2015). DNA and cDNA were further analyzed for cellular p53 by PCR (Malekpour Afshar et al. 2016). JCPyV DNA was detected with a viral load value of 6.0 × 10^4^ gEq/mL. nPCR for LTAg region gave a positive result for the 5ʹ end, whereas sequences encoding the C-terminal region and the *VP1* gene were undetectable. Only LTAg transcripts of 5ʹ were identified. The NCCR had the archetype A-B-C-D-E–F box arrangement. miRNAs’ investigation showed no miR-J1-5p expression. Moreover, p53 analysis showed a negative result at both DNA and RNA level as well as by immunohistochemical analysis.

## Discussion

Viral sequences have been detected in human brain tissue corroborating the hypothesis that JCPyV could be involved in the development of human brain tumors (Ahye et al. 2020). In our study, qPCR showed that PXA, a rare brain tumor with histopathological features resembling PML, harbored JCPyV DNA. To define JCPyV as infectious agent associated with brain cancer, JCPyV DNA positivity alone is not sufficiently specific to establish its etiological role. For this reason, in this study the expression of the *LTAg* and *VP1* at the DNA and RNA level was investigated.

In 1998, Boldorini et al. identified JCPyV LTAg and VP1 DNA sequences, but not virus particles in the brain tissue of a 9-year-old boy with PXA (Boldorini et al. 1998). In our case, we detected DNA sequences and transcripts, corresponding to the 5ʹ end but not the 3ʹ end of the *LTAg* gene and VP1 was undetectable both at DNA and RNA level.

During viral replication, JCPyV displays an orderly gene expression cascade in which *LTAg* transcript is expressed first followed by the expression of the *VP1* gene. Loss of the viral replication capacity is a common feature of virus-associated tumors. In this case, the hampered viral replication could explain the expression of *LTAg* but not *VP1* gene.

Since NCCR and miRNA could represent independent modalities of regulating JCPyV replication at the transcriptional and post-transcriptional levels, in this study, NCCR architecture and miRNA expression were investigated.

We detected a NCCR with archetype architecture and the absence of miR-J1-5p expression. Since JCPyV miRNA-5p has been proposed to act as an important safeguard, reducing *LTAg* expression during viral persistence, in our study we could speculate that the absence of JCPyV-miRNA expression and the detection of LTAg contribute to a persistence state rather than a viral reactivation.

We failed to detect p53 at the DNA, RNA, and protein level in our tumor specimen. As previously described, the C-terminal region of LTAg can interact with p53 (Zheng et al. 2022) although inactivation of p53 is not absolutely required for polyomaviruses to induce cancer as shown for MCPyV and MCC. In fact, the LTAg expressed in MCPyV-positive MCC lacks the p53 binding domain but retains the Rb domain (DeCaprio 2021), as observed in our case. Therefore, further studies are required to establish whether the genesis of PXA could depend on the transformation capacity of LTAg by Rb sequestration, as demonstrated in the MCC. Additional studies are required (i) to establish whether full-length or C-terminal truncated LTAg is expressed; (ii) to establish whether the truncation is the result of a deletion in the LTAg gene rather than nonsense mutation (as is the case in the LTAG gene of MCPyV-positive MCCs); and (iii) to explain the lack of p53 DNA detection in the tumor.

## Conclusion

JCPyV detection in brain tumor tissue reinforces the idea that this virus could be involved in tumor development. However, further studied are warranted to define its role in oncogenesis of brain tumors.

## Data Availability

No datasets were generated during the study.

## Abbreviations

PXA: Pleomorphic xanthoastrocytoma
PML: Progressive multifocal leukoencephalopathy
JCPyV: JC polyomavirus
qPCR: Quantitative polymerase chain reaction
nPCR: Nested polymerase chain reaction
LTAg: Large T antigen
NCCR: Non-coding control region
VP1: Viral protein 1
miRNAs: MicroRNAs
Rb: Retinoblastoma
CNS: Central nervous system
